# A little more conversation – the influence of communicative context on syntactic priming in brain and behavior

**DOI:** 10.3389/fpsyg.2014.00208

**Published:** 2014-03-18

**Authors:** Lotte Schoot, Laura Menenti, Peter Hagoort, Katrien Segaert

**Affiliations:** ^1^Max Planck Institute for PsycholinguisticsNijmegen, Netherlands; ^2^Donders Institute for Brain, Cognition and BehaviourNijmegen, Netherlands

**Keywords:** syntax, syntactic priming, overt production, comprehension, communication, fMRI, behavior

## Abstract

We report on an functional magnetic resonance imaging (fMRI) syntactic priming experiment in which we measure brain activity for participants who communicate with another participant outside the scanner. We investigated whether syntactic processing during overt language production and comprehension is influenced by having a (shared) goal to communicate. Although theory suggests this is true, the nature of this influence remains unclear. Two hypotheses are tested: (i) syntactic priming effects (fMRI and behavioral) are stronger for participants in the communicative context than for participants doing the same experiment in a non-communicative context, and (ii) syntactic priming magnitude (behavioral) is correlated with the syntactic priming magnitude of the speaker’s communicative partner. Results showed that across conditions, participants were faster to produce sentences with repeated syntax, relative to novel syntax. This behavioral result converged with the fMRI data: we found repetition suppression effects in the left insula extending into left inferior frontal gyrus (BA 47/45), left middle temporal gyrus (BA 21), left inferior parietal cortex (BA 40), left precentral gyrus (BA 6), bilateral precuneus (BA 7), bilateral supplementary motor cortex (BA 32/8), and right insula (BA 47). We did not find support for the first hypothesis: having a communicative intention does not increase the magnitude of syntactic priming effects (either in the brain or in behavior) *per se*. We did find support for the second hypothesis: if speaker A is strongly/weakly primed by speaker B, then speaker B is primed by speaker A to a similar extent. We conclude that syntactic processing is influenced by being in a communicative context, and that the nature of this influence is bi-directional: speakers are influenced by each other.

## INTRODUCTION

Although in everyday life, the purpose of using language is to communicate, participants in most psycholinguistic experiments use language devoid of any communicative goal: they speak without addressing someone or listen without being addressed directly. The implicit assumption here is that core language processing in the brain is not influenced by whether or not the speaker or listener is in a communicative context and that therefore, non-communicative language experiments can be used to infer what happens in real-life communicative situations. Although we do not wish to imply that this method is incorrect, it has been repeatedly shown that linguistic and extra-linguistic contextual factors can have a significant influence on core language processes (e.g., Nieuwland and Van Berkum, 2006; Van Berkum et al., 2008; Hanulíková et al., 2012). In the current study, we investigate whether being in a communicative context influences core language processing in the brain. Previous studies have reported that certain social factors, which are inherent to any communicative context, can influence core language processing. For instance, (inferred) speaker characteristics can influence semantic (Van Berkum et al., 2008) and morphosyntactic processing (Hanulíková et al., 2012) during language comprehension. Here, we focus on another important aspect of being in a communicative context: having (and perhaps sharing) the intention to communicate. Having a communicative intention engages certain brain regions which do not show activation for speakers without such an intention (see Willems and Varley, 2010). What has not been investigated yet is whether having a (shared) goal to communicate influences how core linguistic information, such as syntax, is processed in the brain. This is the focus of the present study.

We make use of the phenomenon that speakers tend to repeat syntax across sentences, which is known as syntactic priming or structural persistence (Bock, 1986). A large body of research on this topic shows that syntactic priming is not only reflected in production preferences but also in response latencies and brain activation; compared to production of a sentence that is syntactically different from its preceding sentence, speakers start speaking faster (Smith and Wheeldon, 2000) and show less brain activation (Menenti et al., 2012) when they produce a sentence with repeated syntax. Furthermore, syntactic priming effects are not only found for production, but also for comprehension: listeners expect subsequent sentences to have the same syntax (Branigan et al., 2005; Thothathiri and Snedeker, 2008), and again, less brain activation is needed to comprehend repeated sentence structures than novel sentence structures (Noppeney and Price, 2004; Weber and Indefrey, 2009; Menenti et al., 2011). Of importance for the present study is that syntactic priming effects do not only occur within-modalities (production-to-production or comprehension-to-comprehension priming) but also between modalities – and thus, crucially, between speakers (comprehension to production or production to comprehension priming). Speakers not only repeat their own syntax, but also the syntax of others (Potter and Lombardi, 1998; Branigan et al., 2000; Bock et al., 2007) and they expect others to repeat their own syntactic structures back to them (Ferreira et al., 2012). Similarly, suppressed brain activation is found both within and between speakers, for production and comprehension in the same brain regions (Segaert et al., 2012).

Despite the vast number of studies that report different types of syntactic priming effects, there is no definite answer as to why speakers tend to repeat syntactic structures. Well established accounts of syntactic priming propose residual activation (Pickering and Branigan, 1998) or implicit learning (Chang et al., 2000, 2006) as an underlying mechanism, or a hybrid account with elements of both mechanisms (Reitter et al., 2011). Another proposal is that priming has an important communicative function (Pickering and Garrod, 2004; Jaeger and Snider, 2013). If the latter proposal is true, syntactic priming effects should be influenced by being in a communicative context. To date, however, the nature of this influence remains unclear. In this study, we test two specific hypotheses. Both follow from the hypothesis that communicative context has a top-down influence on syntactic priming effects, but they differ with respect to the nature of this influence. However, we do not claim that these hypotheses are necessarily mutually exclusive.

The first hypothesis is that having a (shared) goal to communicate increases the magnitude of an individual’s syntactic priming effects (Garrod and Pickering, 2009). This hypothesis fits well within the *mutual expectation adaptation* model by Jaeger and Snider (2013). This model centers on the idea that listeners (unconsciously) make predictions about upcoming input in order to process language input efficiently. If the listener’s prediction is wrong, however, more processing is needed to overcome this prediction error (cf. Friston, 2005), which will in turn slow down and/or make comprehension more effortful. Jaeger and Snider (2013) propose that speakers can contribute to the minimization of the listener’s prediction error (and thus their comprehension ease) by aligning what they say to (their beliefs about) what the listener expects them to say. Because a listener generally expects syntactic repetition, the listener’s comprehension is facilitated if speakers indeed repeat syntax. In conversation, therefore, both the speaker and the listener are trying to make information transfer as fast and efficient as possible, by contributing to what Jaeger and Snider (2013) refer to as *mutual expectation adaptation*. Syntactic priming effects are a reflection of this process.

If speakers repeat sentence structures because they (unconsciously) believe this facilitates comprehension for the listener, they should be less likely to do so when it is less urgent to make the listener understand what they are trying to communicate. Similarly, listeners may expect more repetition from the speaker if they know that the speaker wants to convey a message to them (Jaeger and Snider, 2013). There are some studies that seem to provide evidence in favor of this hypothesis, reporting stronger syntactic priming effects as the need for (efficient) communication increases (Branigan et al., 2000; Reitter et al., 2006). However, there are also studies that report no difference (Bock et al., 2007), or seem to point in the opposite direction (Ferreira et al., 2012). None of these studies, however, can provide definite evidence in favor of or against the hypothesis. Either the experimenters varied not only communicative intention, but also other aspects of the task (Branigan et al., 2000; Reitter et al., 2006; Bock et al., 2007), or the task is the same, but communicative intention is manipulated for either the prime or the target but not for both (Branigan et al., 2007; Ferreira et al., 2012). None of these studies have compared syntactic priming effects within the exact same task, while only varying the context (communicative or non-communicative) in which participants perform this task, during both target and prime. Furthermore, although the influence of having a communicative intention may be different during production and comprehension, none of these studies have investigated and compared syntactic priming effects in production as well as comprehension. Here, we do include all these aspects in one study in order to test whether syntactic priming effects in production and/or comprehension increase when interlocutors have a (shared) goal to communicate with each other.

The second hypothesis that we will test here takes into account the fact that syntactic priming magnitude may not (only) be influenced by the speaker’s beliefs about the interlocutor’s expectations, but also by the interlocutor’s actual linguistic behavior: the magnitude of the interlocutor’s syntactic priming effects. Previous studies have repeatedly shown that speakers tend to mimic certain aspects of their interlocutor’s linguistic behavior, such as accent (Giles and Powesland, 1975), speech rate (Webb, 1969, 1972) and speech rhythm (Cappella and Panalp, 1981). Pickering and Garrod (2004) have proposed that this kind of automatic mimicking will lead interlocutors to align their representations at different levels of linguistic processing (in the examples above, alignment will occur at the phonetic level). Alignment at lower levels can then lead to increased alignment at higher levels of processing, with the ultimate goal of achieving alignment at the level of the situation model: speakers’ representations of the situation under discussion. Alignment at this level, Pickering and Garrod (2004) argue, is a prerequisite for successful communication. On their own, syntactic priming effects already reflect speakers’ (unconscious) efforts to align their syntactic representations with the interlocutor by mimicking his or her syntactic structures. Here, however, we hypothesize that how strong these syntactic priming effects are is yet another aspect of linguistic behavior that is unconsciously and automatically mimicked by interlocutors. If we take the predictions of Jaeger and Snider’s (2013) *mutual expectation adaptation* model into account, repetition will only facilitate communication if it is expected by the listener. But how does the speaker know how much repetition the listener expects? One option may be to adapt the amount of repetition to the amount of repetition used by the interlocutor. If this is true, this implies that the magnitude of syntactic priming effects should not be studied from an individualistic perspective. Rather, we should take into account the fact that speakers influence each other. This prediction will be tested in the present study: in addition to comparing priming effects of individual participants in a communicative and a non-communicative context, we correlate the strength of priming effects of two participants within one communicative pair.

We test the two hypotheses outlined above using the results of a syntactic priming study. Participants are assigned to a communicative or to a non-communicative condition. The experimental task is identical in both conditions: participants either have to describe photographs of two persons performing a transitive action (e.g., feeding or serving), or listen to descriptions of these photographs and decide whether the photograph matches the description. The difference between the communicative and non-communicative condition is that only in the communicative context, participants work together with another (naive) participant, whereas in the non-communicative context, participants perform the experiment alone, speaking without addressing anyone and listening to pre-recorded sentences. In the communicative condition, the two participants thus describe the photographs to each other: they share the goal to communicate with each other. This goal is absent the non-communicative condition. Therefore, a comparison between participants in these two conditions provides us with a way to test our first hypothesis: syntactic priming effects are stronger when participants have a (shared) goal to communicate. Because we furthermore aim to compare the influence of communicative context on syntactic priming in production and comprehension, we need to measure syntactic priming effects in the same way for both modalities. This is possible using functional magnetic resonance imaging (fMRI): brain activation related to syntactic processing can be measured in the same regions for production and comprehension. We make use of the fMRI adaptation effect, where the blood oxygen level dependent (BOLD) response in certain regions of the brain is reduced when a sentence structure is repeated (Grill-Spector and Malach, 2001; Ganel et al., 2006; Segaert et al., 2013). Priming effects can thus be measured by looking at the decrease of the BOLD-response for sentences in which syntax is repeated, relative to non-repeated. Since these fMRI adaptation effects can be measured in the same brain regions for syntactic priming in production and comprehension (Menenti et al., 2011; Segaert et al., 2012), they provide us with a good measure to compare the strength of syntactic priming effects in different processing modalities, as well as between contexts (communicative vs. non-communicative).

We only obtained fMRI measurements of one of the participants in the communicative context. Therefore, we cannot use fMRI measurements to test our second hypothesis that the priming effects of one participant are influenced by the priming effects of his or her behavioral partner. However, we did obtain behavioral measurements (speech onset latencies) for both participants in a communicative pair. As said above, speech onset latencies show syntactic priming effects if there are faster speech onsets for target sentences with repeated sentence structure relative to sentences with novel sentence structure. The magnitude of priming effects of each individual participant in the communicative context will be correlated with the magnitude of the priming effects of their conversation partner. This analysis will test whether speakers are indeed influenced by the priming effects of their interlocutor.

Thus, in this study, we investigate whether being in a communicative context, i.e., having – or sharing – the intention to communicate, influences core language processing. Specifically, we wish to empirically test the theoretical proposal that syntactic priming effects are subject to the top-down influence of being in a communicative context. We derived two (not mutually exclusive) hypotheses from this proposal, which we test in the present study. The first hypothesis is that the presence of a communicative context will increase the magnitude of the syntactic priming effects. To test this prediction, we compare syntactic priming effects in overt production (both behavioral – speech onset latencies – and in the brain – fMRI adaptation effects) and comprehension (in the brain) of participants in a communicative vs. a non-communicative context. The second hypothesis is that priming effects of one person are influenced by the priming effects of the other person: if person A accommodates to person B, then person B will accommodate to person A to a similar extent. To test the latter prediction, we correlate (behavioral) priming effects measured during language production of two participants in a communicative pair.

## MATERIALS AND METHODS

**FIGURE 1:**
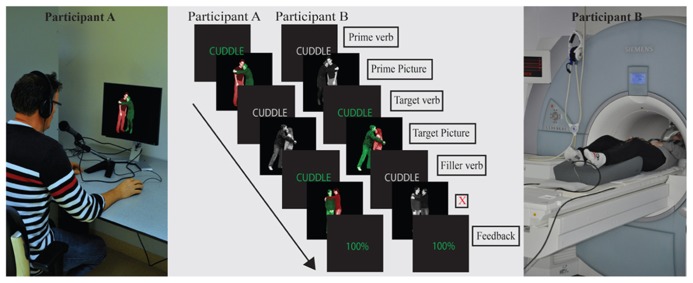
**Set-up of the experiment in the communicative context condition.** Two participants – one in the MRI scanner, one in the behavioral experiment room – describe photographs to each other. (In the non-communicative context, there was only one – MRI – participant.) Trial structure and task were identical in both conditions. Green verbs at the start of a trial indicated that a (color-coded) production photograph would follow; gray verbs indicated a (grayscale) comprehension photograph would follow. Verbs were presented to participants in Dutch (English translation is shown in the figure). Production photographs were color-coded to guide participants’ production: participants were instructed to name the green figure before the red figure, leading them to produce an active or a passive sentence. When participant A in the communicative context produces a description, participant B listens to the description, and *vice versa*. Mismatches in the communicative context were created by showing a different photograph to speaker and listener (in the non-communicative context, a non-matching sentence recording was played to the participant). In both contexts, the listener needs to press a button when a mismatch is noticed. Feedback screens were only present in the communicative context: they reflect the percentage of hits minus false alarms and misses by both participants. Feedback was only presented within a filler block.

For the present report, we collected data from participants who perform a syntactic priming experiment in a communicative context: one participant is in the MRI scanner and the other one performs the experiment in a behavioral experiment room (see **Figure 1**). This dataset could be used to test the (second) hypothesis that priming effects of one person are influenced by the priming effects of their communicative partner. To test the (first) hypothesis that syntactic priming effects are stronger in a communicative context, we compare participants in a communicative context with participants in a non-communicative context. The data on syntactic processing in a non-communicative context were collected before and have already been reported on in Segaert et al. (2012). To be able to compare the two contexts, we kept all aspects of the testing procedure and fMRI data acquisition parameters maximally similar. As a consequence, the experiment in the communicative context was performed as previously described in Segaert et al. (2012) with identical materials and methods. The one crucial difference between the communicative and non-communicative context was that in the non-communicative context, participants performed the experiment alone, whereas in the communicative context, participants worked together with another participant.

### PARTICIPANTS

For 24 participants in the non-communicative condition (12 male, mean age 22 years, SD = 4.8) fMRI (and simultaneously also behavioral) measurements were obtained. In the communicative condition, we paired two participants (one in the MRI room and one only behavioral participant): there were 24 participant pairs (48 participants). The 24 MRI participants in the communicative condition (11 male, mean age 21 years, SD = 2.35) had a similar distribution of sex and age as the 24 participants in the non-communicative condition. The 24 behavioral-only participants in the communicative condition (five male, mean age 20.5 years, SD = 2.37) were not gender matched with either group of MRI participants. Participants pairs in the communicative context condition (one male–male pair, 10 male–female pairs, four female–male pairs and nine female–female pairs) did not know the partner they would cooperate with during the experiment. However, they met each other before entering the experiment room and they interacted during the instructions and sound set-up and during the break. All participants were right-handed native Dutch speakers without neurological or language impairments and with normal or corrected to normal vision. Participants had attended or were attending university education in the Netherlands and gave written informed consent prior to the experiment. They were always compensated for their participation, either financially or through course credits.

### EXPERIMENTAL DESIGN

**FIGURE 2:**
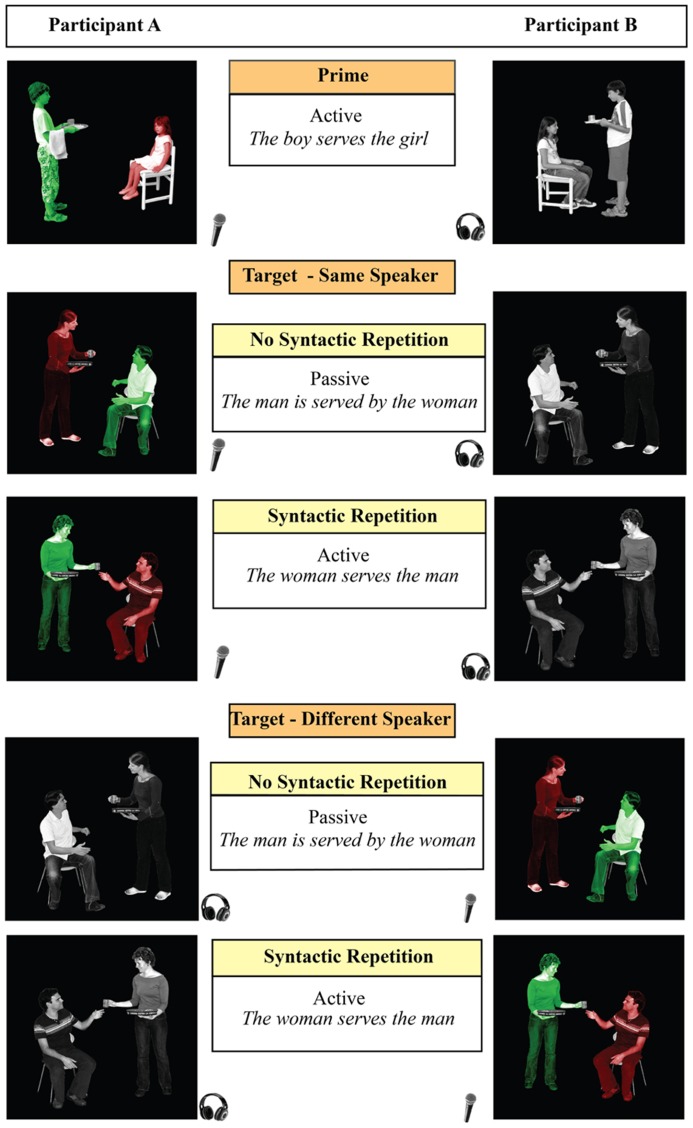
**The design figure illustrates the within-subject factors.** Communicative context was manipulated between subjects (in the communi-cative context, participants A and B speak and listen to each other; in the non-communicative context, there is only one participant). Four within-participant factors are manipulated for each level of the between-participant factor context: Syntactic Repetition (repeated or novel syntax), Speaker Switch (same speaker or different speaker), Target Modality (production or comprehension), and Target Structure (active or passive). The figure illustrates only the first three: between prime and target, syntactic structure (active or passive) and speaker could be the same or different. From the perspective of one participant, the processing modality could thus be repeated or not, with the modality of the target being comprehension or production. In both contexts, materials were presented to participants in Dutch: examples in the figure are translated to English.

Non-communicative context vs. communicative context was a between-participant manipulation (factor Context). Within each level of this factor, the same four within-participant factors were manipulated: Syntactic Repetition (syntax was novel vs. repeated compared to the preceding sentence), Speaker Switch (same speaker vs. different speaker compared to the preceding sentence), Target Modality (participant is the speaker or the listener during the target trial), and Target Structure (active vs. passive voice). This resulted in 16 within-participant conditions. The design (eight conditions resulting from crossing three of the within participant factors, leaving out the within-participants factor Target Structure and the between-participants factor Context) is illustrated in **Figure 2**. Stimuli were presented in a running priming paradigm where each target item also served as the prime sentence for the next target item (see **Figure 1**).

### TASK

The participants’ tasks during production and comprehension trials were identical in the non-communicative and the communicative context. Task-specifications as stated below can therefore also be found in Segaert et al. (2012).

During production trials, the participant’s task was to describe the color-coded photographs overtly with a short sentence using the verb that was presented immediately before the photograph appeared on the screen. Participants were instructed to name the green actor before the red actor (*stop light paradigm*; Menenti et al., 2011). Other than the appearance of the photographs, there was no cue for the participants to start the description; they could freely start whenever they were ready.

During comprehension trials, a sentence-photograph matching paradigm was used (Clark and Chase, 1972). Participants were presented with a photograph and heard a description, either pre-recorded [non-communicative condition; presented following the photograph with an interstimulus interval (ISI) of 0–1000 ms] or provided by the other participant (communicative condition). For more details on the sentence recordings that were used in the non-communicative context, see Segaert et al. (2012). Participants were instructed to press a button whenever the photograph that was presented to them did not match the description they heard.

### STIMULUS MATERIAL

In both the non-communicative and the communicative condition, we asked participants to describe photographs, or to listen to a description of a photograph. We used identical photographs in both conditions. Therefore, the details of stimulus material as described here can also be found in Segaert et al. (2012). All photographs had been pretested previously (Menenti et al., 2011) to establish whether the depicted actions were clear and to measure which verb was most commonly used to describe the action. Due to reasons explained in Segaert et al. (2012), during the experiment this verb was presented immediately preceding the photographs for production and comprehension trials. Participants were required to use that verb in their description of the photograph. For comprehension trials, photographs were accompanied by pre-recorded descriptions in active or passive voice in the non-communicative condition. These recorded descriptions were not used in the communicative condition, as the participants listened to a real time description of the other participant (for details about the recordings see Segaert et al., 2012).

The photographs that were used to create the target trials depicted 36 different events with a patient and an agent performing an action, which can be described with a transitive verb such as “feeding” or “serving.” Each event was enacted by four different couples (two man–woman and two boy–girl couples) and for each couple, there was one photograph with the male and one photograph with the female in the agent role. Furthermore, two photographs were made for every agent-patient combination: one with the agent on the left and one with the agent on the right. This led to 16 different photographs for each event. For each of these photographs, three versions were created to differentiate between comprehension and production targets. For comprehension trials, a grayscale version was shown. For production targets, photographs were color-coded to elicit descriptions in the passive or active voice (see section Experimental Design). The active version of the photograph had a green agent and a red patient, for the passive version the actor is red and the patient is green (see examples in **Figures 1** and **2**).

The filler items were created with a different set of photographs. Filler items were added to provoke variability in the syntactic structures and in the lexical items that participants produced/heard during the experiment. There were photographs depicting one actor performing an action that can be described with an intransitive verb, such as “singing” or “running,” and photographs depicting two inanimate objects or one actor and one inanimate object, the relation between which can be described with a locative verb, such as “standing” or “lying.” Three versions were again created for each photograph: two color-coded versions for the production trials and one grayscale version for comprehension trials. For the intransitive production targets, the actor was shown in green or red. For the locatives, color-coded versions of the photographs were used to elicit a locative state (“the ball lies on the table”) or a frontal locative (“on the table lies a ball”). For intransitives, the actors were sometimes famous people (e.g., former U.S. president Bush), animals, or people that could be named by their profession (e.g., the policeman).

### LIST COMPOSITION

List composition was largely identical in the non-communicative and the communicative condition (details for the non-communicative condition can also be found in Segaert et al., 2012). Participants were presented with 320 target items (20 items in each of the 16 conditions). In addition to this, there were 80 transitive structure items that serve as prime-only items at the beginning of target blocks. This increases the total number of items in target blocks to 400. Target items were presented in 80 blocks with an average length of five transitive structures (range 3–7 items). The verb was always repeated between the items in one target block. The conditions followed each other in a random order that was different for every participant, with two constraints on the order of conditions. The first is that no condition is repeated twice in a row and the second is that a target item with adults is always followed by an item with children and *vice versa*, so that there was no lexical repetition between items other than the verb. In a full list of items presented to the participant, the same action or the same actors could occur several times, but the combination of actors and actions was unique. Target blocks were alternated with filler blocks. Since in target blocks the verb was always repeated between items, the verb was also repeated between filler items within one block. For 10% of the filler items, this was not the case to bring in some extra variation. There were 280 filler items, divided over 80 blocks (2–5 filler items per block, average length of 3.5). Each participant thus received 680 trials in total (320 targets, 80 prime-only and 280 filler trials), which were divided over two scanning sessions (45 min each). Each photograph could occur only once in the experiment and every participant saw a different list of items.

In the non-communicative condition, 10% of the filler items consisted of a mismatch between the photograph that the participant saw and the recorded sentence that the participant heard. For example, while seeing a photograph that depicted a man kissing a woman, the participant could hear: “the man punishes the woman” or “the woman kisses the man.” In these cases, participants had to press a button. In the communicative condition, mismatches were created by showing a different photograph to the participants (see **Figure 1**). Thirty-five percent of the filler items in the communicative context were intended mismatches. Only half of the mismatches in the communicative context (17.5%) needed to be detected by the fMRI participant though (i.e., a mismatch between the fMRI comprehension trials and the behavioral production trials). The other half needed to be detected by the behavioral participant (behavioral comprehension trials – fMRI production trials). This mismatch percentage for the fMRI participant in the communicative condition was increased relative to the non-communicative condition to make the feedback percentages (see below) more variable. For both contexts, there were mismatches between photograph and description for transitive photographs (50% of all mismatches) and intransitive/locative photographs (50% of all mismatches). Additionally, participants in the communicative condition also created their own mismatches when the speaker gave a wrong description of the photograph. No mismatch trials were included in the analyses.

In the non-communicative context, the detection-rate of the mismatches was used to check whether participants pay attention during comprehension: syntactic and semantic processing was necessary to detect these mismatches. In the communicative condition mismatch-trials have an additional function: since it depends on both participants whether the mismatch is correctly detected, the detection-rate is a good measure of how well participants are working together. Mismatches can therefore be used to enhance the feeling of having a shared communicative goal. We increased this feeling in two ways. First, participants heard a beep whenever one of them pressed a button. That way, they both knew a mismatch was detected by the participant that saw a comprehension trial. Second, visual feedback was provided, which showed a percentage that indicated how well participants were performing the task. This percentage was based on the mismatches that were not correctly detected (misses), but also on false alarms: participants pressing the button when there was no mismatch between photographs. Errors can arise due to either participant, the speaker can make a mistake during photograph description; the listener can fail to detect a description mistake or can incorrectly detect a description mistake. Thus, the participants’ joint effort is reflected in the feedback percentages. Participants saw a feedback screen 26 times during the entire experiment. These feedback trials were always presented within a filler block, but not after the final item of this block (i.e., not directly preceding a prime). So, every third filler block participants were presented with feedback.

### TRIAL STRUCTURE AND PROCEDURE

Trial structure was identical in both conditions (see also Segaert et al., 2012, for the non-communicative context only). Each trial started with the presentation of the verb. This verb was color-coded to let the participants know whether a “comprehension photograph” or a “production photograph” would follow. Green verbs preceded production photographs and gray verbs preceded comprehension photographs. When one participant in the communicative condition (fMRI/behavioral) saw a green verb, introducing a production photograph, the other participant saw a gray verb, after which a comprehension photograph would follow. After presentation of the verb (500 ms) and an ISI of 500–2500 ms, a photograph (in color for production trials, gray for comprehension trials) was shown for 2000 ms before the screen turned black.

Before starting the experiment, participants read instructions on paper and the experimenter checked whether they understood everything. In the communicative context condition, the experimenter flipped a coin to decide which of the two participants would perform the experiment in the MRI scanner. We included this procedure to make sure participants were convinced of working with another naive participant, rather than a confederate. Hereafter, one participant was placed in the MRI scanner and the other was installed in a separate, quiet room.

Participants completed a short practice block before the actual experiment started. After the practice trial, they had the opportunity to ask questions. Furthermore, in the communicative context condition, both participants were asked whether they could hear each other well. The experiment consisted of two runs of 45 min, both in the communicative and the non-communicative context. Between the two runs, fMRI participants underwent an anatomical T1 scan. All participants then got a short break outside the MRI scanner/experiment room. After the experiment there was a debriefing during which all participants in the communicative context indicated that they believed that they were interacting with another participant and not a confederate.

### fMRI DATA ACQUISITION

Acquisition parameters in the non-communicative and communicative context condition were identical: this section is therefore identical to the data acquisition section in Segaert et al. (2012). Participants were scanned with a Siemens 3-T Tim-Trio MRI scanner, using a 12-channel surface coil. To acquire functional data, we used parallel-acquired inhomogeneity-desensitized fMRI (Poser et al., 2006). This is a multiecho echo-planar imaging sequence, in which images are acquired at multiple time echoes (TEs) following a single excitation [time repetition (TR) = 2.398 s; each volume consisted of 31 slices of 3 mm thickness with slice gap of 17%; isotropic voxel size = 3.5 × 3.5 × 3 mm^3^; field of view (FOV) = 224 mm). The functional images were acquired at following TEs: TE1 at 9.4 ms, TE2 at 21.2 ms, TE3 at 33 ms, TE4 at 45 ms, and TE5 at 56 ms, with echo spacing of 0.5 ms. This entails a broadened T2^*^ coverage because T2^*^ mixes into the five echoes in a different way, and the estimate of T2^*^ is improved. Accelerated parallel imaging reduces image artifacts and thus is a good method to acquire data when participants are producing sentences in the scanner (causing motion and susceptibility artifacts). However, the number of slices did not allow acquisition of a full brain volume in most participants. We made sure that the entire temporal and frontal lobes were scanned because these were the regions where the fMRI adaptation effects of interest were expected. This meant that data from the superior posterior frontal lobe and the superior parietal lobe (thus data from the top of the head) were not acquired in several participants. A whole-brain high-resolution structural T1-weighted magnetization prepared rapid gradient echo sequence was performed to characterize participants’ anatomy (TR = 2300 ms, TE = 3.03 ms, 192 slices with voxel size of 1 mm^3^, FOV = 256), accelerated with GRAPPA parallel imaging.

### DATA ANALYSIS

#### Behavioral data analysis

The experimenter coded production responses of the participants online for correctness. Target trials were considered for analysis if during both prime and target trial 1) the correct structure was used and 2) both actors were named accurately and the presented verb was used correctly (88.25% of all target trials). To be able to make audible recordings (and for the behavioral participant to be able to hear the fMRI participant), we made use of a noise-cancelation microphone inside the MRI scanner, which filtered out most of the noise made by the scanner. For each trial an individual recording started from the onset of the photograph on the screen. From these recordings, speech onset latencies were automatically determined. First, MRI scanner noise was filtered out by the use of a band pass filter (250–2500 Hz), before smoothing the signal and conversion to *z*-scores. We then set a threshold above which the signal could reliably be identified as speech. The same threshold was used for all sound files. Before analyses, onsets that were smaller than 300 ms were excluded from the raw data (0.07% of all correct target trials). Averages and SD were then calculated per participant per condition. Onsets that were more than 2.5 SD away from this participant by condition mean were excluded from further analysis (1.92% of all correct target trials).

Two analyses were carried out using the speech onset data. The first, between-context analysis was done to test our first hypothesis that syntactic priming effects are stronger in a communicative context. We separated the behavioral and the MRI participants in the communicative context to assess whether MRI and behavioral participants would show identical reaction time patterns. A repeated measures analysis of variance (ANOVA) was carried out using the statistical software package SPSS, with within-participant factors Syntactic Repetition, Speaker Switch, Target Modality and Target Structure, and between-participant factor Group (communicative-behavioral, communicative-MRI, and non-communicative-MRI). The second analysis on the behavioral data concerned our second hypothesis. A within-context correlational analysis was carried out on the syntactic priming effects of the MRI and behavioral participants in the communicative condition, also using SPSS, to see whether priming effects correlate within participant pairs (i.e., between the MRI and behavioral participant). For the latter analysis, we split the priming effects into between-participants (i.e., comprehension to production) priming effects and within-participants (i.e., production to production) priming effects, and performed separate, identical analyses for both datasets. The reason for this split was that if participants indeed accommodate to each other and their priming effects are correlated, this effect will be strongest for between-participant priming, and weaker (or even non-existent) for within-participant priming effects.

#### fMRI data analysis

***Preprocessing***. For both contexts, fMRI data were preprocessed as described in Segaert et al. (2012), using statistical parametric mapping (SPM5) (Friston et al., 2007). The first five images were discarded to allow for T1 equilibration. Then the five echoes of the remaining images were realigned to correct for motion artifacts (estimation of the realignment parameters is done for one echo and then copied to the other echoes). The five echoes were combined into one image with a method designed to filter task correlated motion out of the signal (Buur et al., 2009). First, echo 2–5 (i.e., TE2, TE3, TE4, and TE5) were combined using a weighting vector with the weights depending on the measured differential contrast to noise ratio. The time course of an image acquired at a very short echo time (TE1) was then used in a linear regression as a voxelwise regressor for the other image (i.e., the result of combining TE2, TE3, TE4, and TE5) in the same echo train acquired with high BOLD sensitivity. The resulting images were coregistered to the participants’ anatomical volume, normalized to Montreal Neurological Institute space, and spatially smoothed using a 3D isotropic Gaussian smoothing kernel (full-width at half-maximum = 8 mm).

***Whole-brain analysis***. All fMRI analyses were performed in order to compare participants in the communicative condition with the participants in the non-communicative condition. As said above, the data from the non-communicative context had already been collected for the Segaert et al. (2012) experiment. First- and second-level statistics were performed using the general linear model framework of SPM5 (Friston et al., 2007). One main regressor contained information about the between-participant factor Context (communicative or non-communicative). Within each level of Context there were 16 main regressors coding for the 16 conditions resulting from the 2 × 2 × 2 × 2 design with within-participant factors Syntactic Repetition, Target Modality, Speaker Switch, and Target Structure. An explicit baseline (fMRI measurements during the presentation of verbs) was used. In the first-level linear model, we modeled the individual start time of the photograph during production trials or the start time of the pre-recorded utterance (non-communicative context) or the “live” description (communicative context) during comprehension trials. We modeled the hemodynamic response function only as related to these onsets and set the duration as a constant event. Separate regressors were included for the verbs, photographs during comprehension trials, filler items, items which were only primes, and incorrect responses. The events of the model were convolved with the canonical hemodynamic response function provided by SPM5. Also the temporal derivatives were included in the model. Furthermore, six motion parameters (realignment parameters: translation along, and rotation around, the *x*, *y*, and *z* axes) and two parameters which correct for global intensity fluctuations (compartment signal parameters: white matter and cerebral spinal fluid; Verhagen et al., 2008) were added as regressors. For the second-level random-effects analysis, we used the beta-images of the 16 main regressors for each condition, leading to a total of 32 main regressors in the second level between-context model. The cluster size was used as the test statistic and only clusters significant at *p* < 0.05 corrected for multiple non-independent comparisons are reported. Local maxima are also reported for all clusters with their respective *Z* values.

***Region of Interest analyses***. Two region of interest (ROI) analyses were performed. We opted for this approach because we expect to find differences between participants in the two contexts in regions related to syntactic processing. ROI analyses then allow us to check for interactions with more sensitivity than whole-brain analyses. There were two sets of ROIs. The first set of ROIs corresponded to the activation clusters for which a main effect of Syntactic Repetition was found in the whole-brain analysis. A second ROI-analysis was done based on regions in which significant syntactic priming effects were reported previously for production and comprehension: the left inferior frontal gyrus and in the left posterior middle temporal gyrus (Menenti et al., 2011). For each cluster, average time courses were calculated using Marsbar (). For the ROI analysis at the second level, a repeated measures ANOVA was carried out with the factors Region, Syntactic Repetition, Speaker Switch, Target Modality, Target Structure, and Context on the subject contrast values using SPSS. The aim of both of these analyses was to establish with higher sensitivity whether there were interactions with the factors Syntactic Repetition and Context. Interactions of interest were Syntactic Repetition ^*^ Context (^*^Region) and Syntactic Repetition ^*^ Context ^*^ Speaker Switch (^*^Region). The latter interaction is interesting because the effect of communicative context may be more pronounced for between-speaker priming (Speaker Switch) than for within-speaker priming (No Speaker Switch).

## RESULTS

### TASK PERFORMANCE (ACCURACIES)

Participants from all three groups (fMRI non-communicative – *N* = 24, fMRI communicative – *N* = 24, behavioral communicative – *N* = 24) performed equally well on the production and comprehension task. In the production task, fMRI participants responded correctly on 96% of the trials in the non-communicative context and on 98% of the trials in the communicative context condition. For the comprehension task, the average d-prime for fMRI participants was 0.91 in the non-communicative context condition and 0.88 in the communicative context condition. A *t*-test revealed no difference between the two MRI groups on their performance (*p* > 0.1). For the behavioral participants, the average d-prime was 0.87. Performance of participants within one pair did not differ significantly (*p* > 0.7).

### HYPOTHESIS 1 – IS SYNTACTIC PRIMING STRONGER IN A COMMUNICATIVE CONTEXT? BETWEEN-CONTEXT ANALYSES (NON-COMMUNICATIVE VS. COMMUNICATIVE CONTEXT) IN BEHAVIOR AND BRAIN

In this section, we report the results of the analyses that we did to test the hypothesis that syntactic priming effects are stronger in a communicative context. That is, we compare the magnitude of syntactic priming effects between participants in the non-communicative and the communicative condition. The results of three analyses are reported: one with respect to participants’ behavioral results (speech onset latencies) and two with respect to their brain results (fMRI adaptation effects on the whole-brain and ROI level). For the comparison of behavioral effects, we included all three participant groups (MRI and behavioral participants in the communicative context). For the comparison of syntactic priming effects in the brain, naturally, only the participants in the two MRI groups are taken into account.

#### Behavior (speech onset latencies)

**FIGURE 3:**
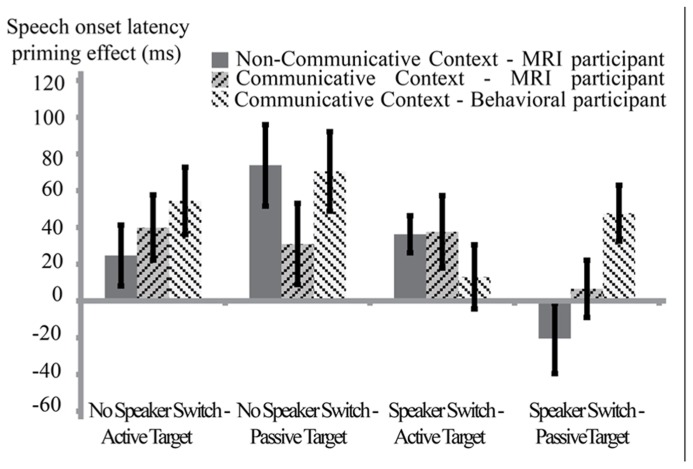
**Between-context analysis (1): behavioral results for three groups of participants.** Speech onset latency-priming effects (novel – repeated syntax) in ms (error bars reflect standard errors), split by Speaker Switch (no speaker switch means production prime – production target; speaker switch means comprehension prime – production target) and Target Structure (active or passive voice). Dark-gray bars with solid fill represent the average priming effect of the MRI participants in the non-communicative condition. Of the two bars with striped pattern fill, the darkest gray bar represents the MRI participants in the communicative context and the lighter gray bar represents behavioral participants in the communicative condition. There were speech onset latency-priming effects in the two communicative as well as in the non-communicative condition. The groups differed from each other in the Speaker Switch – Passive target condition, in that only the behavioral participants in the communicative context showed a significant priming effect here. There was no overall interaction Syntactic Repetition ^*^ Group: it is not the case that participants in the communicative context show stronger syntactic priming effects than participants in the non-communicative context.

In this analysis, we compared behavioral syntactic priming effects of the participants in the communicative context (in the MRI scanner and in the behavioral experiment room) to the syntactic priming effects of participants in the non-communicative context. We ran a repeated measures ANOVA with the factors Syntactic Repetition, Speaker Switch, Target Structure, and Group (communicative-behavioral, *N* = 24 communicative-MRI, *N* = 24 and non-communicative-MRI, *N* = 24). Results from this analysis (see also **Figure 3**) showed a significant effect for Syntactic Repetition [mean_No-Repetition_ = 1065.9 ms, SE = 24 ms, mean_Repetition_ = 1031.3 ms, SE = 23 ms, *F*(1,69) = 30.34*, p* < 0.001], Target Structure[mean_Active_ = 998.4 ms, SE = 22 ms, mean_Passive_ = 1098.9 ms, SE = 26 ms, *F*(1,69) = 126.62, *p* < 0.001], Speaker Switch [mean_NoSwitch_ = 1054.8 ms, SE = 24 ms, mean_Switch_ = 1042.4 ms, SE = 22 ms, *F*(1,69 = 4.01, *p* < 0.05] and Group [mean_Communicative-Behavioral_ = 962 ms, SE = 27 ms, mean_Communicative-MRI_ = 1096 ms, SE = 27 ms, mean_NonCommunicative-MRI_ = 1087 ms, SE = 39 ms, *F*(2,69) = 3.77, *p* < 0.03]. The main effect of Syntactic Repetition indicates that the speech onset latencies show a syntactic priming effect. Crucially, however, there was no two-way interaction between Syntactic Repetition and Group[*F*(2,69) = 0.884, *p* > 0.4]. Results did show a significant interaction between Speaker Switch and Syntactic Repetition [*F*(1,69) = 8.64, *p* < 0.005]. Follow-up tests showed that for all groups, the syntactic priming effect was largest when target and prime were produced by the same speaker. The difference lies in the novel syntax condition. When having produced the prime themselves, speakers are slower to produce a sentence with a novel syntax than when the prime was produced by a different speaker (*p* < 0.01). In the repeated syntax condition, there was no difference between the two speaker switch conditions (*p* > 0.8). There was also a significant four-way interaction between Speaker Switch, Syntactic Repetition, Target Structure, and Group [*F*(2,69) = 3.35, *p* < 0.05]. Follow-up tests on the latter interaction revealed that the three groups differed from each other in the condition where there has been a speaker switch between prime and target, and the target is a passive structure [*F*(2,69) = 4.21, *p* < 0.02]. For both of the MRI groups, there was no effect of Syntactic Repetition in this condition (*p* > 0.05) whereas there was for the behavioral participants in the communicative context (*p* < 0.05).

#### Brain (fMRI adaptation effects)

**FIGURE 4:**
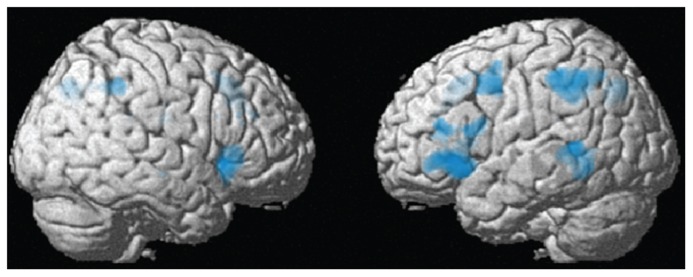
**Between-context analysis (2): whole-brain results (see also Table 1)**. In the left insula extending into left inferior frontal gyrus (BA 47/45), left middle temporal gyrus (BA 21), left inferior parietal cortex (BA 40), left precentral gyrus (BA 6), bilateral precuneus (BA 7), bilateral supplementary motor cortex (BA 32/8), and right insula (BA 47), there was a repetition suppression effect for repeated compared to novel syntactic structures, in the communicative as well as the non-communicative condition.

***Whole-brain analysis***. For the whole-brain analysis, we used an uncorrected voxelwise threshold of *p *< 0.001 and a cluster-level threshold corrected for multiple comparisons of *p *< 0.05. As displayed in **Figure 4** and **Table 1**, there were several regions showing a repetition suppression effect to repeated syntax (conditions with novel syntax minus conditions with repeated syntax): left insula extending into left inferior frontal gyrus (BA 47 and BA 45), left middle temporal gyrus extending into inferior temporal cortex (BA 21 and BA 37), left inferior parietal cortex extending into superior parietal cortex (BA 40 and BA 7), left precentral gyrus (BA 6), bilateral precuneus (BA 7), bilateral supplementary motor area extending into right anterior cingulum (BA 32/8 and BA 32), and right insula (BA 47). These regions are thus less activated for sentences with a repeated syntax than for sentences with novel syntax; they show repetition suppression for syntax. There were no repetition enhancement effects. At the whole-brain level, there were no regions that showed significant interactions between Syntactic Repetition and Context (i.e., more repetition suppression for communicative context) or between Syntactic Repetition, Context, and Speaker Switch (i.e., more repetition suppression for communicative context in the conditions where the prime speaker is not the same as the target speaker; production prime – comprehension target and comprehension prime – production target).

**Table 1:** Whole brain analysis – Results for the main effect of Syntactic Repetition (no syntactic repetition > syntactic repetition) and the interactions Syntactic Repetition * Context and Syntactic Repetition * Context * Speaker Switch.

Anatomical label	BA	Global and local maxima			Cluster-level		Voxel-level
		*X*	*Y*	*Z*	*K*	*P*(corrected)	*Z*
***Main effect Syntactic Repetition (No Syntactic Repetition* > *Syntactic Repetition)***
Left inferior parietal	40	-42	-44	40	928	<0.001	5.37
Left inferior parietal	40	-52	-36	46			4.68
Left superior parietal	7	-32	-62	48			3.54
Left precentral	6	-38	2	44	424	<0.001	5.16
Left precentral	6	-46	0	42			4.30
Left precentral	6	-46	8	42			4.20
Left precuneus	7	-6	-70	40	333	<0.002	5.02
Right precuneus	7	8	-72	40			3.71
Right precuneus	7	14	-58	42			3.56
Left supplementary motor area	32/8	-8	22	46	408	<0.001	4.98
Right supplementary motor area	32/8	6	18	48			4.19
Right anterior cingulum	32	14	36	26			3.33
Left insula	47	-38	20	-6	895	<0.001	5.18
Left inferior frontal pars prbitalis	47	-32	30	-4			4.69
Left inferior frontal pars triangularis	45	-48	34	0			3.85
Left middle temporal	21	-50	-44	2	387	<0.001	4.54
Left middle temporal	21	-54	-46	4			4.33
Left inferior temporal	37	-58	-54	-6			3.64
Right insula	47	36	24	0	452	<0.001	4.98

Syntactic Repetition * Context
No significant clusters

Interaction Syntactic Repetition * Context * Speaker Switch
No significant clusters

*There were no significant interactions with context, indicating that the syntactic priming effects are not stronger in the communicative context than in the non-communicative context.*

***ROI analyses***. To maximize detection power, we also investigated possible interactions between the factors Syntactic Repetition and Context in ROI analyses. The sensitivity on the whole-brain level may have been insufficient to detect interactions with a between-group factor. ROI analyses allow searching for potential interactions between Syntactic Repetition and context at the highest possible statistical sensitivity. Analyses of variance were carried out with the within-participant factors Region, Syntactic Repetition, Speaker Switch, Target Modality and Target Structure and the between-participants factor Context.

The first ROI-analysis included the seven regions that were derived from the clusters that showed significant repetition suppression effects for syntax in the whole-brain analysis reported above. There were no interactions between Syntactic Repetition and Context: the interactions Syntactic Repetition ^*^ Context (^*^Region) and Syntactic Repetition ^*^ Speaker Switch ^*^ Context (^*^Region) were not significant in this analysis (all *p* > 0.1).

**FIGURE 5:**
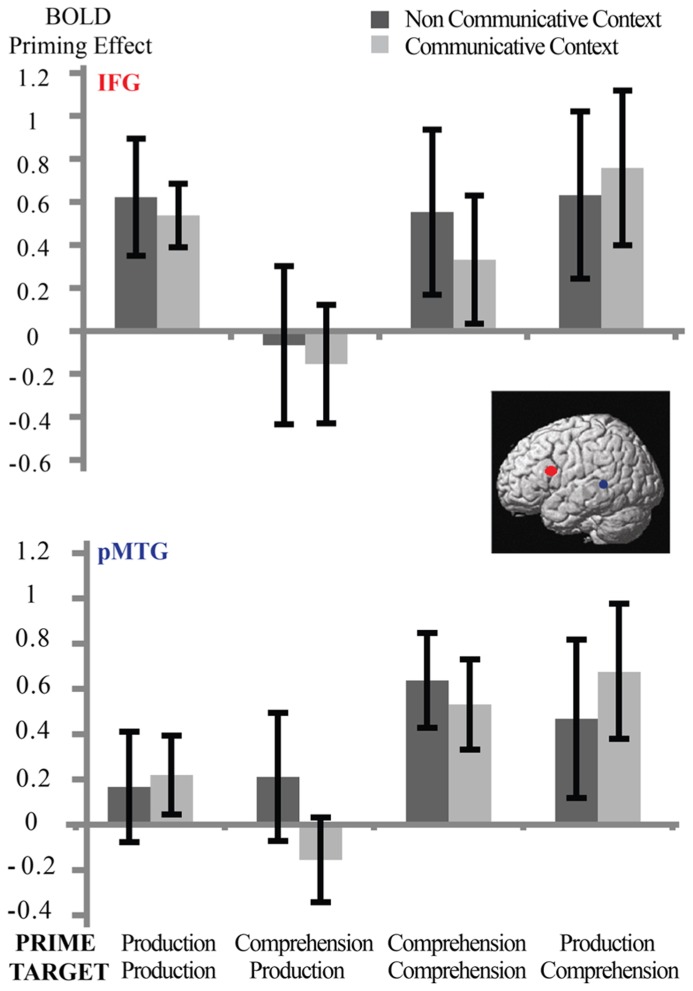
**Between-context analysis (3): ROI-analyses in two clusters based on Menenti et al. (2011): left inferior frontal gyrus (top) and left pMTG (bottom)**. Error bars reflect standard errors. There is a main effect of Syntactic Repetition in both clusters but no interaction with Context: participants in the non-communicative and communicative condition do not differ in the strength of their repetition suppression effects in these regions.

We also performed a second ROI analysis (see **Figure 5**) in two pre-defined regions; the left inferior frontal gyrus and the left posterior middle temporal gyrus (clusters based on Menenti et al., 2011). Although there were significant main effects for repetition in both regions (left inferior frontal gyrus:* p* < 0.01; left posterior middle temporal gyrus: *p* < 0.005), again, there were no significant interactions between Syntactic Repetition ^*^ Context or Syntactic Repetition ^*^ Speaker Switch^*^ Context (all *p* > 0.7). Interactions with repetition that were significant were Target Modality ^*^ Speaker Switch ^*^ Repetition in left inferior frontal gyrus (*p* < 0.02) and Target Modality ^*^ Repetition in left posterior middle temporal gyrus (*p* < 0.02).

In sum, even with the increased detection power of ROI analyses, and in two different ROI analyses, we did not find evidence that the repetition suppression effects for syntactic Repetition differ between the communicative and non-communicative context.

### HYPOTHESIS 2 – iS SYNTACTIC PRIMING IN COMMUNICATION INFLUENCED BY THE INTERLOCUTOR’S BEHAVIOR? WITHIN-CONTEXT (COMMUNICATIVE CONTEXT ONLY) ANALYSIS IN BEHAVIOR

In this section, we report the results of the analysis that we did to test the second hypothesis that the syntactic priming effects of one speaker in a communicative pair are influenced by the syntactic priming effects of the other speaker in that pair. This analysis is done for the participants in the communicative context only: we correlated the behavioral (speech onset latency) priming effects of the MRI and the behavioral participants who were paired.

#### Correlation analysis between two interlocutors in the communicative context

**FIGURE 6:**
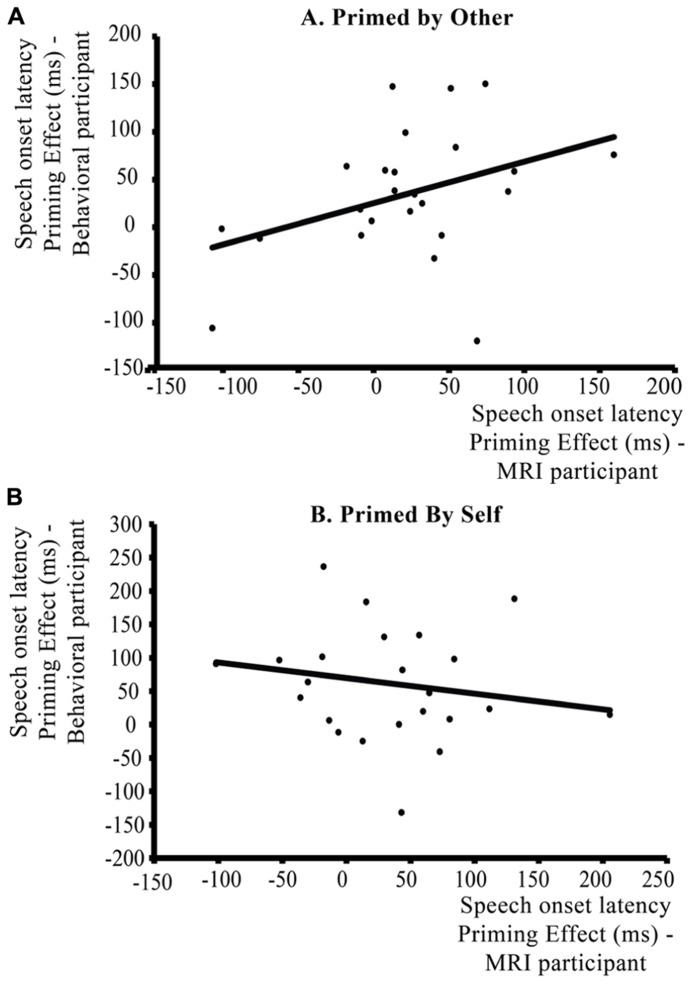
**Within-context analysis: correlation between individual magnitudes of syntactic priming effects (repeated – novel syntax) in speech onset latencies (ms) for the participants in the communicative context.** The *y* axis represents the average syntactic priming effect of the behavioral participant, the *x* axis the average syntactic priming effect of the participant in the MRI scanner. Priming effects are spit according to Speaker Switch: **(A)** shows the correlation between priming effects when participants are primed by their partner (Speaker Switch – comprehension to production priming), **(B)** shows the correlation for trials where participants are primed by themselves (No Speaker Switch/production to production priming). When primed by the other participant, there is a positive correlation between the priming effects of participants in a communicative pair, whereas there is no significant correlation between participants when they are primed by themselves.

There was a significant positive correlation between the average behavioral priming effect (speech onset syntax not-repeated – speech onset syntax repeated) of the MRI participants and the average priming effect of the behavioral participants over trials in which participants were primed by each other [**Figure 6A**: *r* = 0.382, *p *(one-tailed)**< 0.04]. The stronger the priming effects for the MRI participant when the prime is provided by the behavioral participant, the stronger the priming effects for the behavioral participant when the prime is provided by the MRI participant. As a control, this correlation was not significant for the average priming effects over trials where the participants were not primed by the other participant but primed by themselves [**Figure 6B**: *r* = -0.189, *p *(one-tailed) > 0.15]. Thus, when a speaker is primed by another person, the average syntactic priming effect of this interlocutor in the conversational pair is influenced by the average syntactic priming effect of the other interlocutor in that pair.

#### Additional evidence: exploratory analyses

Although the correlation presented above shows that speakers in a communicative pair are influenced by their interlocutor, this correlation is based on individuals’ average syntactic priming effects across the entire experiment. However, if speakers indeed adapt their syntactic priming effects to their interlocutor, it is likely that individual syntactic priming magnitude changes over time. Speakers have to be exposed to their interlocutor’s linguistic behavior (in this case, to their syntactic priming magnitude) before they can adapt their own behavior accordingly. The present experiment was not designed to investigate how syntactic priming effects change over time. However, due to the fact that participants got a break in the middle of the experiment, we could compare speakers’ behavior in two consecutive sessions (i.e., two halves of the experiment). Because we find the correlation only for between-speaker priming, in the exploratory analyses presented below, we only take between-speaker priming effects into account.

**FIGURE 7:**
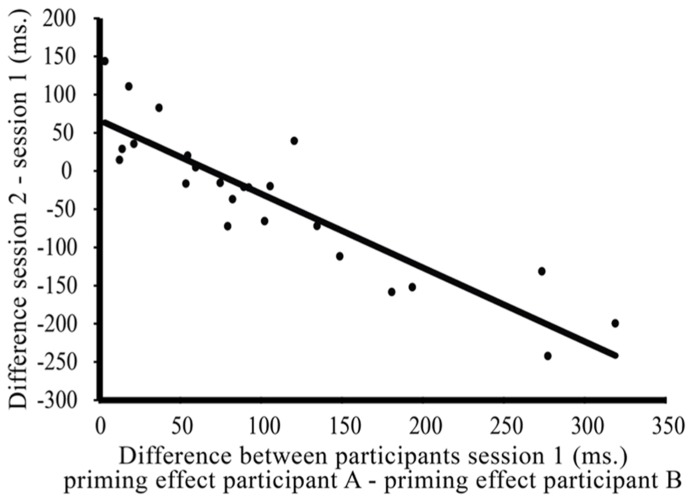
**Correlation between the difference between paired participants’ syntactic priming effects in session one of the experiment (*x*-axis: priming effect speaker A – speaker B) and the decrease/increase of that difference over time (*y* axis: difference part two – difference part one).** So, the more different paired individual’s syntactic priming effects are in session one of the experiment, the more this difference will decrease between session one and session two.

If individual syntactic priming effects change over time with the (unconscious) goal to adapt one’s own priming effects to the interlocutor, we expect that the syntactic priming effects of two speakers in a communicative pair become more similar over time. In other words, we would expect that the* difference* between paired individuals’ syntactic priming effects (priming effect speaker A – priming effect speaker B) decreases over time. Our data seem to be in line with this: an exploratory paired samples *t*-test showed that on average, the difference between paired individuals’ syntactic priming effects decreases between session one (mean difference = 106.13 ms, SE = 17.88 ms) and session two [mean difference = 70.23 ms, SE = 8.83 ms; *t*(23) = 1.85, *p* < 0.08]. Furthermore, we see that the variance between pairs decreases between sessions [*F*(1,46) = 6.68, *p* < 0.02]. So, we do not only see that within pairs, the difference between individuals’ syntactic priming effects decreases between sessions, but also that the variance between pairs – with respect to this difference – decreases. Therefore, we would expect that the strength of the decrease in the difference between individual’s syntactic priming effects will be proportional to how different they are at the start of the experiment. A final, correlational analysis (see **Figure 7**) provides further support for this: the more different syntactic priming effects of individuals in a communicative pair are at the start of the experiment (here: session one), the more this difference will decrease over time (here: between session one and session two; *r* = -0.891, *p *< 0.001).

## DISCUSSION

In this study, we investigated whether syntactic processing during overt production and comprehension is subject to the top-down influence of being in a communicative context, i.e., having or sharing the intention to communicate. Specifically, we examined whether communicative context influences the magnitude of syntactic priming effects. Before addressing this issue, though, we first established that there were syntactic priming effects both in behavior and in the brain in both the non-communicative and communicative context. In behavior we found that speakers are faster to start producing sentences with a repeated syntax compared to sentences with a novel syntax. In the brain we found that during production as well as comprehension brain activation is suppressed for sentences with repeated syntax compared to sentences with novel syntax (i.e., repetition suppression) in regions associated with syntactic processing and its downstream consequences [left insula extending into left inferior frontal gyrus (BA 47/45), left middle temporal gyrus (BA 21), left inferior parietal cortex (BA 40), left precentral gyrus (BA 6), bilateral precuneus (BA 7), bilateral supplementary motor cortex (BA 32/8), and right insula (BA 47)]. We then tested two specific hypotheses with regards to the nature of the influence of communicative context on the magnitude of syntactic priming effects. Our first hypothesis was that priming effects are a way for speakers to adapt to the needs and expectations of their conversational partners. If so, the presence of a communicative context should increase syntactic priming effects: if you want to communicate something, you are more likely to adapt to the other person than if you do not have such an intention. To test this prediction, we compared the syntactic priming effects of participants in a communicative context (i.e., two participants addressing each other) to the effects of participants doing the same experiment in a non-communicative context (i.e., speaking without having a direct addressee and listening without being addressed directly). Both with respect to behavior (speech onset latencies) and brain activations (repetition suppression effects on whole-brain and ROI-level), our results did not support the first prediction: participants did not show stronger syntactic priming effects in a communicative context. We did find support for the second hypothesis we put forward: the magnitude of speakers’ syntactic priming effects is influenced by the magnitude of the priming effects of their interlocutor. The correlation we found between individual between-speaker syntactic priming effects of two participants within one communicative pair showed that their syntactic priming magnitudes are related: if participant A is strongly/weakly primed by participant B, then participant B is also be strongly/weakly primed by participant A.

The absence of evidence in favor of our first hypothesis should be interpreted with caution, like any null-result should. Below, we consider some aspects of our design that may have confounded our results and obscured the difference between priming effects in the non-communicative and the communicative condition. First, however, we will discuss the outcomes of our analyses in more detail to get a better understanding of whether the results we do observe are in line with previous studies.

In behavior we found that syntactic repetition speeds up production. This is in line with previous reports on syntactic priming in production latencies (Smith and Wheeldon, 2000; Corley and Scheepers, 2002; Wheeldon and Smith, 2003; Segaert et al., 2011; Wheeldon et al., 2011). We furthermore observed that the behavioral syntactic priming effects were stronger in the within-participant priming condition (no speaker switch between prime and target) than in the between-participant priming condition (speaker switch between prime and target). These findings are in line with results from a corpus study by Gries (2011) who reports that speakers’ tendency to repeat syntax increases for within-speaker priming, relative to between-speaker priming. We also observed that for the speaker switch condition, the syntactic repetition effect for passives depended on whether the participant that produced the target was performing the experiment lying in the MRI scanner (in the non-communicative or communicative context) or not (behavioral participants in the communicative context). Only the participants in the communicative-behavioral condition showed syntactic priming effects for these particular targets, whereas the two MRI groups did not. Although we have no definite explanation as to why the two groups of MRI participants did not show a significant syntactic priming effect for passives when a speaker switch has taken place, literature on syntactic priming effects in production latencies has shown that this type of syntactic priming effect is more reliably found for actives than passives [see Segaert et al. (2011) for an account].

Our neuroimaging results also closely relate to the literature on syntactic priming and syntactic processing. As syntactic priming facilitates syntactic processing, we expected a modulation of the BOLD-reponse in syntactic processing areas. Indeed, of the brain regions in which repetition suppression effects were found, the left inferior frontal gyrus and left middle temporal gyrus are considered core syntactic processing areas (Indefrey et al., 2001; Haller et al., 2005; Snijders et al., 2009; Menenti et al., 2012; Griffiths et al., 2013). The other regions that showed significant repetition suppression effects in our study are not always considered to be core regions in the syntactic processing network, but all of these individual regions have been found to be activated together with the left inferior frontal gyrus and left middle temporal gyrus in studies aimed at identifying the syntactic processing network: left inferior parietal cortex (Haller et al., 2005; Menenti et al., 2012) left precentral gyrus (Menenti et al., 2012), bilateral precuneus (Segaert et al., 2013), bilateral supplementary motor cortex (Segaert et al., 2012), and the right insula (Haller et al., 2005). Therefore, we feel assured that we are looking at the syntactic processing network and its downstream consequences in the human brain.

Due to the fact that our analyses do show syntactic priming effects in behavior and in the brain which are in line with the literature, we feel confident that the absence of evidence in favor of a modulation by communicative vs. non-communicative context is not a fluke. However, we do acknowledge that some aspects of our experimental design may have obscured the difference between the non-communicative and the communicative context.

Firstly, theories proposing that syntactic priming has a communicative function (Pickering and Garrod, 2004; Jaeger and Snider, 2013) refer to speakers’ production choices for a particular syntactic structure relative to a constructional alternative. In our experiment, however, we did not give speakers a choice between syntactic structures. The reason for this was that for reliable fMRI analyses, many trials are needed for each condition. This number is much higher than the occurrence of passives in a free-choice experiment. Therefore, we opted for the design described above. However, we believe that this did not affect our results, as we do find significant priming effects in this type of design, both in behavior and in the brain. Moreover, we find a top-down effect of communicative context on the magnitude of these priming effects, as evidenced by the correlation between the magnitude of syntactic priming effects of two participants in a communicative pair. Therefore, we believe that the lack of difference between participants in the communicative and the non-communicative context is not due to the way we opted to measure syntactic priming effects.

Second, we may not find a difference between syntactic priming effects in a non-communicative and a communicative context because the difference between these contexts may not have been strong enough. Several factors may have contributed here. One is that the recordings that were used in the non-communicative context condition were as natural as possible. Perhaps a less natural, more computerized recording could have increased the difference between contexts and thus could have influenced the magnitude of priming effects. Another factor is that it might be possible that the participants may have unconsciously considered the experimenter to be their addressee in the production conditions. Participants were told by the experimenter that she would listen to what the participant was saying as the fMRI room and experimenter room are connected through an intercom system. If the participants addressed their speech to the experimenter, participants in both groups have a direct addressee. As we intended to manipulate communicative context by the presence or absence of an addressee, this may have obscured our effect. As a last factor that may have decreased the difference between communicative and non-communicative context, we consider the possibility that although the participants in the communicative context condition met each other before the experiment started and were encouraged to interact during technical set-up, they might have forgotten they were actually working together with this other participant during the experiment. However, we do not believe this is the case: although participants could not see each other during the experiment, they could indeed hear each other. Furthermore, during the break in the experiment, participants saw each other again and almost always spontaneously started talking about their performance on the task. Their conversations showed that they were aware that the percentage that was shown to them during feedback trials reflected their joint performance: before returning to their separate rooms for the second half of the experiment, participants said things like: “*this time let’s go for 100% correct!*” Finally, the correlation between individual between-speaker priming effects of conversation partners indicates that speakers are indeed influenced by their conversational partner. We found that if speaker A adapts to speaker B, speaker B adapts to speaker A to a similar extent. This result indicates that priming effects are influenced by being in a communicative context: this influence does not seem to be reflected in an increase of syntactic priming magnitudes *per se*, but rather by the fact that speakers can be influenced by the priming effects of their interlocutors.

The fact that we found a correlation between the magnitudes of syntactic priming effects of conversation partners suggests that syntactic priming should not only be studied as an individualistic phenomenon but rather that both interlocutors should be taken into account. In the non-communicative context, we see that every individual speaker has a different susceptibility to syntactic priming: some speakers show strong syntactic priming effects, whereas other speakers do not. However, the correlation between the magnitudes of syntactic priming effects of individual speakers in a conversation pair shows something which determines the syntactic priming strength above and beyond speakers’ individual susceptibility to priming: the magnitude of one speaker’s priming effects is influenced by the interlocutor’s priming magnitude. This finding is in line with other studies that have shown a tendency for speakers to mimic certain aspects of their interlocutor’s linguistic behavior (Webb, 1969, 1972; Giles and Powesland, 1975; Cappella and Panalp, 1981). The exact mechanism through which this occurs is subject to further research. Our exploratory analyses already seem to indicate that syntactic priming effects change over time, so that speakers in a communicative pair become more similar to each other. Also, the more different syntactic priming effects of individuals in a communicative pair are at the start of the experiment, the more this difference will decrease over time. However, in the exploratory analyses we reported, syntactic priming effects were compared between two sessions. In future studies, we plan to look at change over time more carefully, and define the priming effect at the start of the experiment on the basis of a separate pre-test in which the participants are not influenced by their interlocutor. These future investigations will also investigate the directionality of the adaptation process. The present analyses can only tell us that there is at least one speaker who adapts his or her syntactic priming effects to the interlocutor. In future research, we would like to investigate whether both speakers move toward each other and end up exactly in the middle between their individual priming susceptibility, or whether one speaker could be influenced more than the other. Previous research has identified several social factors that may explain why individuals are more or less primed by their conversation partner. On the one hand, specific characteristics of an addressee seem to influence a speaker’s syntactic priming effects. If these characteristics are valued positively by the speaker, syntactic priming effects are stronger (Balcetis and Dale, 2005). On the other hand, there are also characteristics of the speaker that may play a role in one’s susceptibility to syntactic priming: Weatherholtz et al. (2012; submitted) found that speaker’s strategy to manage conflict mediates the strength of syntactic priming effects (speakers who compromise during conflict repeat syntax more often than speakers who do not comprise).

We conclude that syntactic processing is subject to the top-down influence of being in a communicative context. We did not find evidence in favor of the hypothesis that the presence of a communicative context increases syntactic priming effects *per se*. Rather, the evidence we report here supports the hypothesis that communicative context influences priming effects in that speakers are influenced by each other. This indicates that it is informative to not only study syntactic priming from an individualistic perspective, but rather take the syntactic priming effects of both interlocutors into account.

## Conflict of Interest Statement

The authors declare that the research was conducted in the absence of any commercial or financial relationships that could be construed as a potential conflict of interest.
